# Investigating the neurobiology of maternal opioid use disorder and prenatal opioid exposure using brain organoid technology

**DOI:** 10.3389/fncel.2024.1403326

**Published:** 2024-05-15

**Authors:** Ila Dwivedi, Gabriel G. Haddad

**Affiliations:** ^1^Department of Pediatrics, School of Medicine, University of California, San Diego, La Jolla, CA, United States; ^2^Department of Neurosciences, School of Medicine, University of California, San Diego, La Jolla, CA, United States; ^3^Rady Children’s Hospital, San Diego, CA, United States

**Keywords:** brain organoids, brain spheroids, induced pluripotent stem cells, opioid use disorder, prenatal opioid exposure, opioids

## Abstract

Over the past two decades, Opioid Use Disorder (OUD) among pregnant women has become a major global public health concern. OUD has been characterized as a problematic pattern of opioid use despite adverse physical, psychological, behavioral, and or social consequences. Due to the relapsing–remitting nature of this disorder, pregnant mothers are chronically exposed to exogenous opioids, resulting in adverse neurological and neuropsychiatric outcomes. Collateral fetal exposure to opioids also precipitates severe neurodevelopmental and neurocognitive sequelae. At present, much of what is known regarding the neurobiological consequences of OUD and prenatal opioid exposure (POE) has been derived from preclinical studies in animal models and postnatal or postmortem investigations in humans. However, species-specific differences in brain development, variations in subject age/health/background, and disparities in sample collection or storage have complicated the interpretation of findings produced by these explorations. The ethical or logistical inaccessibility of human fetal brain tissue has also limited direct examinations of prenatal drug effects. To circumvent these confounding factors, recent groups have begun employing induced pluripotent stem cell (iPSC)-derived brain organoid technology, which provides access to key aspects of cellular and molecular brain development, structure, and function *in vitro*. In this review, we endeavor to encapsulate the advancements in brain organoid culture that have enabled scientists to model and dissect the neural underpinnings and effects of OUD and POE. We hope not only to emphasize the utility of brain organoids for investigating these conditions, but also to highlight opportunities for further technical and conceptual progress. Although the application of brain organoids to this critical field of research is still in its nascent stages, understanding the neurobiology of OUD and POE via this modality will provide critical insights for improving maternal and fetal outcomes.

## 1 Introduction

As of 2023, opioids persist as the group of substances with the greatest contribution to the global burden of disease, given the significance of their physical, psychological, behavioral, social, and economic impact across wide ranging demographics (UNODC World Drug Report, 2023). Opioids are synthetic or semi-synthetic substances with substantial pharmaceutical value, due to their ability to alleviate chronic pain and promote sedation. However, their tendency to produce euphoria and high levels of positive reinforcement has also conferred these drugs with significant misuse liability, which has been a driving force behind the opioid epidemic (Strang et al., 2020).

A population that has remained especially vulnerable to the epidemic is pregnant women, with rates of prescription and illicit opioid use and misuse during pregnancy rising steadily over the past two decades (Patrick et al., 2012; Desai et al., 2014; Krans and Patrick, 2016; Krans et al., 2016b; National Academies of Sciences, Engineering, and Medicine et al., 2017; Ko et al., 2020). This unregulated consumption of opioids has led to simultaneous escalations in the prevalence of maternal Opioid Use Disorder (OUD) (Haight et al., 2018; Hirai et al., 2021), a continuum of symptoms manifesting as increased drug cravings and tolerance, physical or psychological dependence, and eventual addiction (Dydyk et al., 2024). Consequently, there has also been a parallel upswing in mothers seeking Medication-Assisted Treatment (MAT) for OUD via the clinical application of opioid-based pharmacotherapies like methadone, buprenorphine, and/or naltrexone, which competitively block euphoria induced by other opioids, prevent withdrawal symptoms, and reduce the risk of overdose or relapse (Krans et al., 2019).

Rising rates of maternal OUD and MAT have fostered interest in the neurobiological etiology and effects of the disorder, as well as the impact of opioid exposure on the developing fetal central nervous system (CNS). Concerns regarding the latter, in particular, have grown due exogenous opioids’ ability to cross the placenta and accumulate in fetal and neonatal tissues (de Castro et al., 2011; Kongstorp et al., 2019; Rosenfeld, 2022). Including these insights into prenatal opioid pharmacokinetics, much of what is currently known regarding the neurobiology of OUD and prenatal opioid exposure (POE) has been gleaned from preclinical studies in animals as well as postnatal or postmortem clinical investigations in humans. Both OUD and POE have long been associated with neurocognitive deficits (i.e., in learning, memory, and attention) and neuropsychiatric co-morbidities (i.e., anxiety, mood disorders like depression, PTSD, etc.) in animals as well as humans (Brooner et al., 1997; Conway et al., 2006; Farid et al., 2008; Ross et al., 2015; Herlinger and Lingford-Hughes, 2022; Balalian et al., 2023). Human neuroimaging studies have further linked both conditions to microstructural gray and white matter disruptions across several brain regions (Radhakrishnan et al., 2021; Herlinger and Lingford-Hughes, 2022). Furthermore, our current understanding of the neurocircuitry of opioid addiction, which spans the mesocorticolimbic system, largely stems from animals (Feltenstein and See, 2008; Strang et al., 2020), while information about OUD heritability primarily comes from human genome-wide association and eQTL studies (Levran et al., 2012; Gelernter et al., 2014; Hancock et al., 2015; Nielsen et al., 2015; Jensen, 2016; Strang et al., 2020). Alterations potentially underlying the cognitive effects of POE have also been primarily gleaned from murine models, including wide-ranging perturbations in neuronal and glial genesis, growth, morphology, maturation, proliferation, plasticity, and function (Ross et al., 2015; Hauser and Knapp, 2018).

To date, these explorations have provided substantial insight into the neurobiological causes and neurological consequences of maternal OUD and POE. However, the nature of their study subjects (human and animal alike) has complicated the interpretation of these findings. Translation of results from animal studies is challenging, given species-specific differences in behavior, neurodevelopmental trajectories, cellular diversity, opioid receptor expression patterns, and opioid pharmacokinetics/bioavailability (Semple et al., 2013; Ross et al., 2015; Marshall and Mason, 2019). Although such issues are eliminated in human subjects, the postnatal or postmortem status of these individuals introduces a new set of confounding factors. For instance, variations in postnatal subject age, individual and maternal health, concomitant exposure to multiple drugs, nutrition, and even socioeconomic background may affect study outcomes (Ross et al., 2015; Radhakrishnan et al., 2021). Experiments using postmortem samples may be additionally problematic because of discrepancies in causes of death, tissue collection methods or timing, and length of sample storage. Importantly, ethical and logistical considerations have also limited the availability of fetal tissues, posing a significant technical obstacle for examinations of POE.

Brain organoid technology has enabled researchers to circumvent the dual challenge posed by confounding experimental variables and sample inaccessibility that has plagued prior studies. Brain organoids are 3D self-aggregating cellular structures that recapitulate key aspects of the human brain’s development, structure, and function, in a region-specific or non-specific manner. These cultures are generated from embryonic (ESCs) or induced pluripotent stem cells (iPSCs) in a process that retains patient genetic backgrounds and permits genetic modification. As they develop and mature, organoids also provide unique access to the brain’s molecular and cellular heterogeneity, pattering, connectivity, and function (e.g., tissue-specific cellular lamination, synapse formation, neurotransmission, and neuronal and neural network activity). Such characteristics, in turn, facilitate the spatio-temporal observation of morphological, electrophysiological, transcriptional, proteomic, and/or metabolomic perturbations caused by disease or exogenous drug exposure during brain development (Lancaster et al., 2013; Trujillo and Muotri, 2018; Willner et al., 2021).

In recent years, brain organoids have emerged as a powerful tool for modeling and studying the etiology and progression OUD as well as the consequent effects of POE. In this review, we endeavor to synthesize the advancements in brain organoid technology that have contributed to our understanding of (a) the neurobiology of OUD and (b) the neurodevelopmental impact of prenatal exposure to opioids. We will also reference the 2D iPSC-derived neuronal cultures that have laid a technical or conceptual foundation for the establishment of more complex 3D models of OUD or POE. Through this review, we aim to emphasize the merits of organoid technology for recapitulating the neurobiology of OUD and POE as well as the necessary avenues for expansion in this crucial field of research.

## 2 Brain organoid and spheroid models of opioid use disorder

Despite the heightened prevalence and severity of OUD relative to other neuropsychiatric and substance use disorders, research using brain organoid cultures to model and study this condition has been surprisingly sparse (McNeill et al., 2020; Niemis et al., 2023). Nevertheless, several advances have been made in recent years with regard to recapitulating and exploring the cellular and molecular neurobiology of OUD *in vitro*. In this section, we endeavor to bring these methodological developments, and the insights they provide into opioid dependence, to light (Table 1).

**Table 1:** *In vitro m*odels of opioid use disorder.

Model type	Reference	iPSC somatic origin	Differentiated neuron type or brain region	Opioid(s) studied	OUD subject history and number (*n*)	OUD subject gender (F, M)	Polymorphism studied	OUD-specific
								Aim/Aspect studied
2D	Sheng et al. (2016a)	Skin fibroblasts	Midbrain DA neurons	NA	Opioid-dependent (*n* = 2)	NR	NA	Genetic etiology and DA pathway role
	Sheng et al. (2016b)	Skin fibroblasts	Midbrain DA neurons	NA	Opioid-dependent risk gene carriers (*n* = 2)	F, M (*n* = 1,1)	*hDAT* 3′ VNTR	Genetic etiology and DA pathway role
					Halikere et al. (2020)				Lymphocytes	Inhibitory (iN) neurons	DAMGO, Morphine	Risk gene carriers (*n* = 7)	F, M (*n* = NR)	*OPRM1* (MOR) A118G SNP	Genetic etiology and DA disinhibition role
	Chen et al. (2022)	NR	Neurons (Type NR)	NA		Heroin-dependent (*n* = 60)	M (*n* = 60)	NA						Exosomal miRNA biomarkers of opioid withdrawal	
	Guo et al. (2023)	NR	Pre-BötC neurons	Fentanyl, Codeine, DAMGO, and Methadone	NA (*n* = NR)	NR	NA	Opioid overdose-induced respiratory depression
	Mendez et al. (2023)	Postmortem skin fibroblasts	Cortical neurons	Morphine	Opioid overdose (*n* = 1)	F (*n* = 1)	NA	Testing fidelity of *in vitro* OUD neuronal models
	3D	Boutin et al. (2022)	NR	Cortical spheroids	Drugs targeting opioid-receptors or OUD-psychiatric sequelae	NA (*n* = NR)	NR	NA	Testing compounds to help model or screen for OUD
		Strong et al. (2023)	Neurons and astrocytes	PFC and VTA spheroids	DAMGO	NA (*n* = NR)	F, M (*n* = 1,1)	NA	Region-specific functional effects of OUD intoxication and withdrawal
Ho et al. (2022)		PBMCs	Forebrain organoids (and Forebrain neurons)	Oxycodone and buprenorphine	Opioid use disorder (*n* = 6)	F, M (*n* = 3,3)	NA	Opioid-specific transcriptional effects in OUD brains

Summarization of studies that have generated or utilized 2D neuronal and 3D organoid/spheroid models of OUD. Subject-specific history of opioid dependence is listed as reported in the cited studies. Wherever possible, gender information was included for OUD subjects or for subjects from which OUD models were derived. For each reference, the primary OUD-related purpose or aim is also provided. DA, Dopaminergic; F, Female; *hDAT*, Human dopamine transporter gene; *OPRM1*, Mu-Opioid Receptor 1 gene; M, Male; MOR, Mu-Opioid Receptor; NA, Not applicable (i.e., not relevant to the study); NR, Not reported; PBMC, Peripheral blood mononuclear cell; PFC, Pre-frontal cortex; Pre-BötC, Pre-Bötzinger complex; VNTR, Variable number tandem repeats; VTA, Ventral tegmental area.

### 2.1 Foundational 2D neuronal models of opioid use disorder

Efforts to model OUD *in vitro* began with the generation of iPSC-derived 2D neuronal cultures from individuals either with opioid dependence or carrying genetic variants linked with increased opioid addiction risk. These studies were a response to the lack of patient- and gene-specific research into processes that bring about vulnerability to opioid dependence. The neuronal cultures themselves were patterned to represent cell types relevant to the neuropathology of OUD (Table 1).

In the first such investigation of its kind, Sheng et al. (2016a) generated iPSC-derived midbrain dopaminergic (DA) neurons from opioid-dependent subjects, motivated by the DA system’s association with reward and addiction. Expanding the use of this culture system in a parallel study, they also derived DA neurons from opioid-dependent individuals carrying variable number tandem repeat (VNTR) polymorphisms in the human dopamine transporter (*hDAT*) gene (Sheng et al., 2016b) associated with substance misuse (Heinz and Goldman, 2000). Relative to non-dependent controls, DA neurons from opioid-dependent subjects in both studies exhibited reduced expression of the dopamine D_2_ receptor (*Drd2*). In addition, Sheng et al. (2016b) identified that increased VNTR length corresponded to lower *DAT* transcript levels, implying a role for this polymorphism in the regulation of *hDAT* gene expression. Interestingly, both *Drd2* and *hDAT* expression levels were rescued by treatment with valproic acid (VPA), an anti-epileptic drug implicated in relapse prevention (Romão et al., 2022). Overall, the fidelity of these results to known DA pathway disruptions in OUD (Koob and Volkow, 2016; Burns et al., 2019) and prior neuroimaging studies of OUD patients (Wang et al., 1997; Volkow et al., 2004), reinforced the utility of opioid-dependent subject derived neurons for further studies of opioid dependence and treatment. These initial experiments by Sheng et al. (2016a,b) also highlighted the genetic tractability of iPSC-derived neuronal systems for the study of OUD, opening avenues for further examinations of underlying molecular dynamics through the modification or correction of disease-causing mutations (e.g., via CRISPR).

The use of the paradigm established by Sheng et al. (2016a,b) was next expanded by Halikere et al. (2020) to understand how molecular disruptions upstream of the DA system might enhance susceptibility to OUD. Evidence that DA neurons are excited through the suppression of inhibitory neurons following μ-opioid receptor (MOR) activation, prompted the team to explore the cellular repercussions of disrupting this pathway. To this end, Halikere et al. (2020) derived inhibitory neurons (iN) from individuals carrying the addiction-risk associated A118G single nucleotide polymorphism (SNP) in MOR. In these iNs, MOR activation by the μ-opioids DAMGO or morphine resulted in heightened inhibition, which manifested as reductions in synaptic release. This suppression of iN activity, in turn, implied increases in downstream DA activation and release, as occurs during acute opioid intoxication (Koob and Volkow, 2016; Uhl et al., 2019). Together, these findings constituted novel conceptual progress in understanding the etiology of opioid dependence at a cellular level and provided further rationale for generating OUD-relevant cell types *in vitro.*

While Sheng et al. (2016a,b) and Halikere et al. (2020) focused on understanding the role of gene variants in the pathogenesis of OUD, recent *in vitro* studies have shifted to modeling phases of the opioid addiction cycle (i.e., binge, withdrawal, and anticipation) and/or outcomes like overdose. In a cross-sectional study of heroin-dependent patients undergoing drug detoxification, Chen et al. (2022) used iPSC-derived neurons to ectopically express exosomal miRNAs identified in their blood during various stages of opioid withdrawal. The circulating miRNAs served as biomarkers for OUD progression and also influenced transcriptional programs associated with neurotransmitter dynamics, neurite outgrowth, and neural growth at a cellular level (Chen et al., 2022). The following year, Guo et al. (2023), developed a model of opioid overdose by generating iPSC-derived neurons representing the preBötzinger Complex (preBötC), a brainstem structure necessary for inspiratory rhythm generation, which is suppressed by opioids. These neurons exhibited dose-dependent cessations in activity due to four μ-opioids (fentanyl, codeine, DAMGO, and methadone) and recovery upon naloxone administration (Guo et al., 2023). Although the cells in both studies were not derived from OUD patients, they helped to demonstrate the utility of 2D neuronal cultures for parsing the cellular and molecular changes associated with specific phases of the addiction cycle, as well as identifying valuable biomarkers and therapeutic targets for its sequelae.

Altogether, such rapid developments in the generation of iPSC-derived neuronal models of OUD provoked questions regarding their fidelity to *in vivo* signatures of the disorder. To address this, Mendez et al. (2023), engineered novel iPSC-derived cortical neurons from skin fibroblasts of individuals who had died of an opioid overdose. Following chronic treatment with morphine, these neurons remarkably showed transcriptional alterations paralleling those observed in the postmortem, *ex vivo* frontal cortex tissue of individuals with OUD (Mendez et al., 2023). These included developmental and synaptic genes associated with substance use disorders (Gallo et al., 2018; Seney et al., 2021), as well G-protein-coupled receptor (GPCR) pathways, of interest given that opioid receptors are GPCRs themselves. While there are caveats related to extrapolating disease signatures from postmortem samples (e.g., cellular and molecular deterioration), these findings helped configure an informed, preliminary picture of how efficacious iPSC-derived neuronal cultures can be for recapitulating key molecular features of OUD.

### 2.2 Advancements in brain organoid and spheroid models of OUD

Although Mendez et al. (2023) assembled a strong case for the utility of iPSC-derived neuronal cultures in modeling and studying OUD, their study also highlighted pitfalls associated with their simplicity. Due to their two-dimensional growth patterns, these neurons lack the requisite interactions between heterogenous cell types, multi-dimensional cell–cell contact and communication, and nutrient/oxygen diffusion that confer relevance to *in vivo* neurophysiology. This lack of tissue complexity and organization impacts cellular growth, development, and survival, which complicates interpretations of disease mechanisms (Centeno et al., 2018; Mendez et al., 2023; Mendez and Walss-Bass, 2024). It was these technical gaps in 2D neuronal culture that spurred attempts to recapitulate OUD via 3D brain organoids or spheroids (Table 1).

The inaugural steps toward this objective were taken by studies testing the efficacy of 3D neural spheroids as a high-throughput screening (HTS) platform for compounds intended to model, diagnose, or treat OUD. Despite their limited structural organization compared to organoids, spheroids (self-assembled spheres of different neural cell-types) were chosen for HTS due to their shorter incubation times and relatively higher homogeneity. Boutin et al. (2022) used cortical spheroids to test a library of neuroactive compounds targeting opioid receptors or psychoactive compounds linked with depression, anxiety, and analgesia, which are sequelae of long-term opioid misuse. Following drug exposure, activity changes in these spheroids, represented by fluctuations in calcium fluorescence, were measured using a fluorescent imaging plate reader (FLIPR). MOR agonists were found to have an inhibitory effect, reducing the count and increasing the spacing of calcium activity peaks (Boutin et al., 2022). The consistency of this response with the MOR-activation-induced suppression of cortical neuron activity and synaptic loss in animal studies, demonstrated the potential of this culture system for modeling OUD (Chang et al., 1997; Robinson and Kolb, 1999).

This prospect enabled Strong et al. (2023) to expand the utility of this methodology beyond drug screening to disease modeling. Their team generated novel iPSC-derived neural spheroids mimicking the prefrontal cortex (PFC) and ventral tegmental area (VTA), key regions involved in opioid addiction. Importantly, these spheroids reproducibly retained cell-type compositions that conferred both physiological relevance and region specificity. Considering the vital role of neuronal-glial interactions in neural communication, all spheroids were generated using 90% neurons and 10% astrocytes. Neuronal subtypes in PFC and VTA spheroids were also included in ratios that corresponded to postmortem examinations of the human brain and resulted in unique calcium activity phenotypes. Using this system, Strong et al. (2023) were able to model regional responses to both the intoxication and withdrawal phases of OUD via chronic treatment with and deprivation of the MOR agonist DAMGO. During chronic treatment, PFC-like spheroids experienced reductions in calcium activity peak counts, while treatment and withdrawal both increased peak count in VTA-like spheroids. Although the PFC deficits were rescued by naloxone, the same was not true for the VTA spheroids, indicating fundamental differences in recovery from opioid exposure between brain regions (Strong et al., 2023). This study introduced the first intentional iPSC-derived 3D model of OUD *in vitro*; its value reinforced by the mechanistic insights it provided into region-specific responses to chronic opioid exposure. Moreover, Strong et al. (2023) contributed technical advancements that will prove valuable for future studies of OUD *in vitro*. These included the successful incorporation of genetically encoded biosensors for continuous neuronal activity monitoring in spheroids, and the fusion of VTA- and PFC-like spheroids into assembloids with functional neural circuitry that can be altered by designer drugs.

Contemporary with the development of the neural spheroid model of OUD, Ho et al. (2022) generated the first 3D organoid model of this disorder. Given the PFC’s role in drug reward, withdrawal and relapse during addiction, the group generated iPSC-derived forebrain organoids from individuals with OUD. Subsequently, they used this model to examine mechanisms of differential drug action in opioid-dependent subjects at a single-cell level. Focusing specifically on oxycodone and buprenorphine, two of the most prescribed opioids in the United States, Ho et al. (2022) conducted single-nucleus RNA-sequencing (snRNA-seq) and found that both drugs alter the expression of distinct genes and molecular pathways. While buprenorphine selectively influenced transcriptional regulation in glia, oxycodone activated immune-response associated signaling (STAT1 and type 1 interferon) across several neural cell types in OUD-derived forebrain organoids (Ho et al., 2022). Not only did this research establish a brain organoid model of OUD pathophysiology, but it also established a preliminary repository of drug- and cell-type specific molecular changes associated with opioid-exposure in dependent subjects that may be used for further mechanistic probing or therapeutic development.

Although limited, the technical and conceptual progress made with regard to modeling OUD via 3D organoid and spheroid cultures has been promising. These initial studies have established a strong framework upon which further innovations and mechanistic investigations in the field of addiction research may be conducted *in vitro*. This is especially vital when it comes to maternal OUD, which has not yet been modeled or explored using iPSC-derived neurons, organoids, or spheroids. Prior evidence suggests that drug metabolism and pharmacokinetics are significantly altered by pregnancy and can vary considerably between individuals (Farid et al., 2008; Costantine, 2014; Feghali et al., 2015), which makes the development of patient-, tissue-, gene-, and cell type-specific *in vitro* models even more critical. As these methodologies continue to evolve, their application toward understanding cellular and molecular mechanisms of opioid dependence and addiction during pregnancy will be of prime importance for the improvement of maternal and fetal health, and the identification of novel clinical interventions.

## 3 Brain organoid models of prenatal opioid exposure

While brain organoid models of adult OUD are limited, the technology has been frequently employed in recent years to study the effects of opioids on fetal neurodevelopment. This focus has, in part, been informed by the transcriptional, epigenetic, organizational, and functional correspondence of iPSC-derived neuron and neural tissue maturity to embryonic or fetal brain development (Lancaster and Knoblich, 2014b; Camp et al., 2015; Trujillo and Muotri, 2018; Trujillo et al., 2019; Burke et al., 2020; Mendez et al., 2023). As a result, these cultures have provided unique access to key cellular and molecular features of neurodevelopment in the context of prenatal opioid exposure (POE). In this section, we detail advancements made with regards to modeling and dissecting the neurophysiological and biological effects of opioids on the fetal brain *in vitro* (Table 2).

**Table 2:** *In vitro* models of prenatal opioid exposure.

Model type	Reference	iPSC somatic origin	Differentiated neuron type or brain region	Opioid(s) studied	Subject gender and number (*n*)	Purpose of study
2D	Ju et al. (2021)	Urine exfoliated renal epithelial cells	Neurons (Type NR)	NA	NR (*n* = 6)	Generate MOR and KOR expressing neurons for future studies of opioid-effects.
	Nimbalkar et al. (2023)	Fibroblasts	Nociceptors	DAMGO	M (*n* = 1)	Generate functional nociceptor-mea integrated system for non-opioid analgesic screening.
	Deng et al. (2023)	Umbilical cord blood and fibroblasts	Nociceptors	NA	F, M (*n* = 1,5)	Generate peptidergic and non-peptidergic nociceptors for non-opioid analgesic screening.
	Röderer et al. (2023)	NR	Nociceptors	Fentanyl, dynorphin, and enkephalin	F, M (*n* = 1,1)	Generate functional, opioid-responsive nociceptors for analgesic drug discovery.
3D	Wu et al. (2020)	Fibroblasts	Cortical organoids	Methadone	F, M (*n* = 1,2)	Study methadone’s effect on intrinsic neuronal activity during cortical development.
	Yao et al. (2020)	NR	Cortical organoids	Methadone	NR (*n* = 1)	Study methadone’s effect on developmental neural growth, survival, activity, and communication.
	Dwivedi et al. (2023)	Fibroblasts	Cortical organoids	Methadone	M (*n* = 3)	Study methadone’s transcriptional effect in the developing fetal cortex.
	Nieto-Estévez et al. (2022)	NR	Cortical and subpallial spheroids	Buprenorphine	F, M (*n* = 1,1)	Study buprenorphine’s effect on cortical neural network development (i.e., interneuron migration).
	Fernandes et al. (2022)	NR	Cerebral organoids	Buprenorphine	NR (*n* = NR)	Explore effects of stem cell secretome on opioid receptor activity & neuroplasticity.
	Yao et al. (2023)	Fibroblasts	Cortical organoids	Buprenorphine, Methadone	M (*N* = 1)	Examine differential effects of buprenorphine & methadone on neural growth & network activity.
	Notaras et al. (2021)	NR	Dorsal forebrain organoids	Endomorphin	F, M (*n* = 2,4)	Identify neurodevelopmental signatures of narcotic & neuropsychiatric-risk linked compounds.
	Kim et al. (2021)	Fibroblasts	Midbrain organoids	Fentanyl	NR (*n* = 1)	Examine the cell-type specific transcriptional effects of fentanyl in the developing midbrain.

Summary of all studies to date that have generated *in vitro* models of neurodevelopmental exposure to opioids. Also included are *in vitro* models that will contribute to studies of POE in the future, even if their aim was not directly related. In addition, we have attempted to briefly recap the primary objective of each study, to highlight their respective contributions to the field. F, Female; M, Male; NA, Not applicable (i.e., not relevant to the study); and NR, Not reported.

### 3.1 Technical contributions of 2D neuronal models of prenatal opioid exposure

In contrast to *in vitro* models of OUD, iPSC-derived 2D neuronal cultures used to study the effects of opioid exposure were developed contemporaneously with organoid models of POE. Given this, they cannot strictly be considered foundational for the development of more complex 3D culture systems in this field. Additionally, the usual aim of these studies was not to model POE, but to engineer neural cell types relevant for screening therapeutics that confer neuroprotection or non-opioid based analgesia (Table 2). Therefore, we will only briefly touch upon their findings, focusing instead on the technical advancements that make these cultures pertinent to investigations of POE *in vitro.*

The first neuronal cultures relevant to the study of POE were developed by Ju et al. (2021), who used iPSCs to generate neurons expressing μ-(MOR) and κ-opioid receptors (KOR). Opioids bind to three major opioid receptors throughout the central and peripheral nervous systems: mu-(MOR), kappa (KOR), and delta (DOR). Early preclinical studies of nervous system opioid pharmacodynamics revealed the broad distribution these receptors throughout the CNS, and highlighted differences in receptor expression between fetal/neonatal and adult brains (Barg and Simantov, 1989; Rius et al., 1991; Wittert et al., 1996; Zhu et al., 1998). MORs and KORs are the first opioid-receptors to appear in the fetal brain, while DORs appear postnatally (Rius et al., 1991; Zhu et al., 1998). This finding highlights the possibility that opioids may have distinct effects depending on developmental stage, making the MOR and KOR expressing neurons generated by Ju et al. (2021) an exceedingly relevant model system for POE. In addition, because these neurons originate from exfoliated renal epithelial cells in urine, they afford a unique level of scalability based on the ready supply of source material that can be clinically and non-invasively obtained from maternal OUD subjects or opioid-exposed neonates (Ju et al., 2021).

One problem, however, was that the neurons generated by Ju et al. (2021) did not possess any regional or subtype identity. Consequently, recent efforts have focused on generating cell types more specific to mechanisms of opioid action *in vivo*. Given the broad use of opioids for pain management, Deng et al. (2023), Nimbalkar et al. (2023), and Röderer et al. (2023) worked on generating and modifying iPSC-derived sensory nociceptive neurons as an experimental platform for screening alternative analgesics. Nociception is the process of communicating electrical impulses generated by noxious stimuli. It is important to note that while fetal nociceptive pathways are thought to develop by as early as 7–10 weeks of gestation, the inception of pain sensation or perception remains a controversial topic (Thill, 2022). Therefore, conservatively, these cultures provide a unique opportunity to explore the onset and mechanisms of fetal nociceptive responsivity to opioids. Specifically, the utility of such iPSC-derived sensory nociceptors is conferred by their expression of opioid-receptors (MOR, KOR, DOR, as well as the nociception opioid peptide receptor, NOP) and activity suppression upon μ-opioid exposure (Nimbalkar et al., 2023; Röderer et al., 2023). However, the timing of opioid receptor expression and opioid responsivity varied between protocols. In Nimbalkar et al. (2023), only MOR and KOR were expressed by day 21 in culture, but not DOR. Meanwhile, all opioid receptors were expressed after 21 days of differentiation in the Röderer et al. (2023) study, although signaling through these receptors was not noted until day 70. These differences highlight the caveat of variability that may arise with the use of iPSC-derived *in vitro* models for the study of POE (Volpato and Webber, 2020; Beekhuis-Hoekstra et al., 2021; Brunner et al., 2023; Nath et al., 2023).

Nevertheless, the integration of iPSC-derived nociceptors with multi-electrode arrays (MEA) (Nimbalkar et al., 2023) and the generation of peptidergic and non-peptidergic sensory neurons (Deng et al., 2023) in these studies have expanded the utility of this model for studying opioid effects on electrophysiology and cellular subtypes in the fetal brain. The longitudinal investigation of iPSC-derived nociceptor maturity by Röderer et al. (2023) also supplied helpful information regarding windows of opioid-responsivity in these cultures, with fentanyl only inhibiting the activity of protein kinase A-II (required for pain sensitization) after 70 days of differentiation. Therefore, even though such 2D neuronal cultures have not yet provided insights into the neurodevelopmental effects of POE, they remain advantageous, well-studied platforms upon which future studies may be established.

### 3.2 Brain organoid models of prenatal exposure to methadone

With respect to the development of 3D organoid models of POE, progress has primarily centered around studying the neurodevelopmental impact of opioid-based pharmacotherapies clinically recommended for the Medication-Assisted Treatment (MAT) of maternal OUD (Substance Abuse and Mental Health Services Administration, 2016; The American College of Obstetricians and Gynecologists, 2017) (Table 2). This is due to the rising rates of pregnant women seeking treatment for OUD (Martin et al., 2015; Krans et al., 2019), motivated by the need to improve their own health and prevent neonatal opioid withdrawal (Cleveland and Bonugli, 2014; Frazer et al., 2019; Macfie et al., 2020).

Since the 1970s, MAT using methadone, a synthetic opioid analgesic and full μ-opioid receptor agonist, has been primary standard of care for opioid-addiction during pregnancy (Payte, 1991; Center for Substance Abuse Treatment, 2005; Farid et al., 2008; Krans et al., 2019). However, evidence of methadone’s ability to readily cross the placenta and accumulate in animal and human fetal tissues (Farid et al., 2008; de Castro et al., 2011; Kongstorp et al., 2019; Badhan and Gittins, 2021), in addition to its association with long-term neurocognitive deficits (Wong et al., 2014; Bier et al., 2015; Chen et al., 2015; Hauser and Knapp, 2018; Grecco et al., 2021; Levine et al., 2021; Lum et al., 2021), have led to concerns regarding its effects on fetal neural development *in utero.* These apprehensions have been compounded by methadone’s tendency to cause Neonatal Abstinence Syndrome (NAS), a collection of symptoms associated with withdrawal from POE, which yields CNS hyperirritability and autonomic nervous system dysfunction (Jones et al., 2010; Gaalema et al., 2012).

It was these contraindications that prompted Wu et al. (2020) and Yao et al. (2020) to generate the first iPSC-derived organoid models of POE. These studies integrate human iPSC derived-cortical organoid (hCO) cultures (Trujillo et al., 2019) with immunofluorescence, MEA, or patch-clamp electrophysiology techniques to probe how methadone alters neural growth and function in the embryonic brain. Yao et al. (2020) observed that methadone dose- and timeline-dependently alters the growth of hCOs, while also having a significant effect on neuronal and neural network function. Methadone suppressed the firing of spontaneous action potentials by hCOs attached to MEA plates, which the group hypothesized was likely due to the drug’s concurrent reduction of synaptic transmission (i.e., diminished frequency and amplitude of spontaneous excitatory post-synaptic currents) and voltage-dependent sodium currents that support the initiation of action potential burst firing (Yao et al., 2020).

While Yao et al., 2020 examined the first 3-month of hCO culture, Wu et al. (2020) extended this timeline to track the electrophysiological consequences of methadone exposure in 3–6 month-old hCOs, a period corresponding to neuronal and network activity maturation *in utero* (Trujillo et al., 2018, 2019). They uncovered that 12-weeks of chronic exposure to methadone suppresses the maturation of neuronal membrane properties and excitability via the impairment of voltage-dependent ion channel functions (Wu et al., 2020). Combined with Yao et al. (2020)’s results, these findings provided strong evidence that prenatal methadone exposure causes delays in the onset and progression of neural maturation in the fetal cortex. A subsequent study conducted by Dwivedi et al. (2023) contributed further proof of this effect. Bulk mRNA-sequencing of 2-month-old hCOs that had been chronically treated with methadone for 50 days yielded a robust transcriptional response, pointing toward interrelated alterations in functional components of the synapse, underlying extracellular matrix (ECM), and cilia. Methadone’s impact on molecular processes of synaptic assembly and activity during synaptogenesis in hCOs reinforced the drug’s deleterious influence on neuronal communication and, therefore, maturation of cortical functions (Dwivedi et al., 2023).

Taken together, Wu et al. (2020) and Yao et al. (2020) constituted the first proof-of-concept studies for using brain organoids to study the neurodevelopmental effects of opioids. Alongside Dwivedi et al. (2023), the findings from these investigations provided valuable insights into the structural and functional impact of methadone on fetal cortico-genesis. More specifically, they also supplied the first cellular- and molecular-evidence that methadone impacts synaptogenesis and synapse biology in the human fetal brain. The results from all three papers have helped to initiate a broad picture of how prenatal methadone exposure may give rise to long-term neurologic deficits.

### 3.3 Brain organoid and spheroid models of prenatal exposure to buprenorphine

Alongside methadone, buprenorphine is another widely employed opioid-based pharmacotherapy for the treatment of maternal OUD (Substance Abuse and Mental Health Services Administration, 2016; The American College of Obstetricians and Gynecologists, 2017). The use of this drug during pregnancy has become progressively more common, in part due to its inherent pharmacology (Zedler et al., 2016; Krans et al., 2016a). Unlike methadone, buprenorphine’s nature as a partial MOR and NOP receptor agonist and KOR antagonist with low intrinsic activity means that it can be administered by outpatients with lower overdose risk and fewer drug interactions (Suarez et al., 2022). Moreover, several randomized controlled trials have demonstrated that buprenorphine yields better neonatal outcomes than methadone (Fischer et al., 2006; Kakko et al., 2008; Lacroix et al., 2011; Metz et al., 2011), including fewer signs of NAS and less time or morphine required to treat the syndrome (Jones et al., 2010). Studies in both animals and humans have also shown that prenatal exposure to buprenorphine generates superior neurocognitive outcomes, birth weights, head circumferences, and risks of preterm birth than methadone (Coyle et al., 2012; Zedler et al., 2016; Kongstorp et al., 2019, 2020; Kinsella et al., 2022; Suarez et al., 2022; Gottlieb et al., 2023). Despite these advantages, however, buprenorphine readily crosses the placental barrier (Nanovskaya et al., 2002) and has been linked to adverse postnatal behavioral sequelae (Hung et al., 2013; Sundelin Wahlsten and Sarman, 2013; Tobon et al., 2019), cellular-level alterations in neurogenesis (Pettit et al., 2012; Wu et al., 2014), and deficits in myelination (Sanchez et al., 2008; Eschenroeder et al., 2012). It is these contradictory consequences associated with prenatal buprenorphine exposure that have driven recent efforts to mechanistically dissect its neurodevelopmental effects using 3D organoids.

In 2022, Nieto-Estévez et al. took the first step in this regard by exposing iPSC-derived human cortical spheroids (hCS) and subpallial spheroids (hSS) (expressing markers of developing excitatory and inhibitory interneurons, respectively) to buprenorphine. Their study aimed to dissect the effects of buprenorphine on the crucial yet precarious excitation/inhibition balance that belies cortical network activity (Nieto-Estévez et al., 2022). It was also the first investigation of its kind to use assembloids (Bagley et al., 2017; Birey et al., 2017), fusions of region-specific organoids or spheroids, in order to investigate POE *in vitro*. Upon fusing the hCS and hSS, Nieto-Estévez et al. (2022) observed increased inhibitory interneuron migration from subpallial to cortical spheroids as well as an increase in network activity in response to chronic buprenorphine treatment. Although this latter result seems contradictory, evidence suggests that the inhibitory neurotransmitter GABA exerts an excitatory influence during embryonic development (Leinekugel et al., 1999) that may impact synapse formation and function (Wolf et al., 1986; Caillard et al., 1999a,b). Taken together, these findings suggests that buprenorphine influences both the development, spatial organization, and activity of inhibitory neurons in the cortex.

Interestingly and in contrast to the iPSC-derived neuronal and cortical organoid models of POE cited above, the hCS or hSS generated by Nieto-Estévez et al. (2022) did not express the major opioid receptor subtypes MOR, KOR, or DOR. Buprenorphine was instead found to bind and signal through the NOP receptor, an opioid G-protein coupled receptor expressed throughout the human fetal cortex that does not respond to opioids with known misuse liability (Neal et al., 2002; Zaveri, 2016). Dysregulation of NOP signaling has been linked to psychiatric disease, depression, and memory deficits (Wang et al., 2009; Post et al., 2016; Khan et al., 2018), all of which are sequelae associated with POE. While this feature enabled the team to study buprenorphine’s effects on the fetal brain via the NOP receptor, it limits the use of this model in future studies intending to investigate the drug’s action through canonical opioid receptors.

Unlike Nieto-Estévez et al. (2022), the iPSC-derived cerebral organoids (CeO) generated by Fernandes et al. (2022) were confirmed to express MOR, DOR, and KOR within 30 days of differentiation in both neurons and glial cells. The expression of opioid receptors on glial cells in this model was unique, given discrepancies in findings regarding the presence of these receptors on astrocytes *in vivo* (Stiene-Martin et al., 2001; Machelska and Celik, 2020). Moreover, this was first use of a region non-specific organoid to study POE *in vitro* (Fernandes et al., 2022). Using this model, Fernandes et al. (2022) found that modulating opioid receptor activity using buprenorphine increased apoptosis, astrogliogenesis, glial cell maturation, and dopamine release in CeO cultures, consequences of chronic opioid exposure that have been observed in prior studies. In parallel, the team also subjected their CeOs to the bone marrow stem cell secretome (BMSCSec), which has been explored as an antinociceptive treatment alternative to opioid-based analgesia (Brini et al., 2017; Gama et al., 2018; Khatab et al., 2018). Interestingly, the BMSCSec almost exactly mirrored buprenorphine’s effects, apart from increasing dopamine release. On top of highlighting the effects of buprenorphine on glia during development, the use of CeOs and the stem cell secretome in this study also constituted a technical advancement for POE research. While CeOs provide a novel platform to investigate opioid effects in neurons and glia across developing brain regions (Lancaster and Knoblich, 2014a), the secretome introduces a new method of modulating opioid receptor activity that can be used to individuate the prenatal effects of opioids.

This aim of delineating the effects opioid-based pharmacotherapies was carried forward by Yao et al. (2023), who used iPSC-derived cortical organoids to identify how buprenorphine and methadone differentially affect cellular growth and neuronal activity in the developing cortex. In this study, buprenorphine was found to have a milder effect than methadone on neural growth and activity in hCOs. Although 5–10 times less buprenorphine is required to achieve withdrawal relief than methadone, even at equivalent concentrations buprenorphine did not suppress neural network action potential firing rates. In fact, pre-treatment of hCOs with buprenorphine consistently blocked the severe growth suppressive effects of methadone and the drug even enhanced growth at higher (10 μM) concentrations. Yao et al. (2023) proposed that these distinct effects of methadone and buprenorphine on growth and neural activity are due to their contrasting activity at κ-opioid and NMDA receptors, respectively. Buprenorphine’s antagonism of KOR activity, which has been implicated in cell proliferation, differentiation, and death, as well as its lack of NMDA receptor antagonism were proposed to be the underlying cause of its tempered influence on hCO growth and function (Yao et al., 2023). Cumulatively, these results bring to light novel mechanistic details that may help to explain buprenorphine’s long-supposed superiority when it comes to neonatal outcomes.

### 3.4 Additional brain organoid models of prenatal opioid exposure

As the above-cited articles demonstrate, investigations into prenatal opioid exposure have predominantly been geared toward exploring the effects of opioids used to treat OUD. However, two recent studies exemplify a potential shift in focus toward other opioids as well (Table 2). In a broad exploration of how narcotic and neuropsychiatric-risk factors influence neurodevelopment, Notaras et al. (2021) exposed iPSC-derived forebrain organoids to a panel of “enviromimetic” chemicals and analyzed downstream alterations in transcription, proteomics, and metabolomics. Exposure to opioids was modeled using the endogenous MOR agonist endomorphin, which is central to nervous system pain relief and stress response pathways (Fichna et al., 2007). Interestingly, endomorphin elicited proteomic changes associated with axon guidance, cellular stress response, and RNA regulation, paralleling the effects of cannabinoids, nicotine, and ethanol. Metabolic analyses also revealed converging effects of all treatments on L-Phenylalanine and GTP expression, pointing toward increases in systemic stress (Fernandes et al., 2010) and disruptions in energetics (Leone et al., 2010; Montenegro-Venegas et al., 2010) during corticogenesis. Altogether, this study contributed to the growing body of knowledge surrounding the influence of MOR activation on normative cortical development. Importantly, it also opened a door for future comparative and/or synchronous *in vitro* explorations of opioids and other neurodevelopmentally noxious factors (Notaras et al., 2021).

Nevertheless, endomorphin is not an exogenous opioid, and there are no indications that the neuropeptide’s mimetics are misused during pregnancy. This gap was remedied in a contemporaneous study by Kim et al. (2021), who conducted single-cell RNA-sequencing of iPSC-derived midbrain organoids exposed to fentanyl. Fentanyl is a potent opioid analgesic that is prescribed for the management of severe pain in preterm neonates (Hall and Shbarou, 2009) and during pregnancy, most often during labor (Shoorab et al., 2013). However, fentanyl and its derivatives retain a high degree of misuse potential. As a stark reminder of this fact, fentanyl accounted for 39% of drug overdose deaths in the United States in 2017 (Hedegaard et al., 2019). Therefore, Kim et al. (2021)’s novel examination of fentanyl’s effects on the human fetal midbrain was particularly timely. Corroborating the opioid-induced dysregulation of midbrain dopamine reward pathways (Wei et al., 2018), the group found that acute fentanyl exposure increased dopamine release in the organoids. In contrast, chronic fentanyl treatment arrested the fate determination of neural progenitor cells and altered the expression of synaptic activity and neuronal projection pathways (Kim et al., 2021). These findings were analogous to the neurodevelopmental effects of methadone and buprenorphine reported in cortical or cerebral organoids (Wu et al., 2020; Yao et al., 2020, 2023; Fernandes et al., 2022; Nieto-Estévez et al., 2022; Dwivedi et al., 2023). All the same, this study by Kim et al. (2021) unfurled a list of novel possibilities when it came to studies of POE in organoids, especially regarding the opioid-types and brain-regions modeled.

## 4 Conclusion and future directions

Rising rates of maternal OUD and commensurate fetal opioid exposure have made the development and application of *in vitro* models for both conditions increasingly imperative. Within the past decade, a great deal of progress has been made with regard to recapitulating the neurobiology of OUD and prenatal opioid exposure using 3D brain organoid technology. OUD-specific brain organoids or spheroids have provided valuable insight into the disorder’s genetic etiology, neural mechanics, and downstream neurobiological effects (Table 1). Likewise, subjecting region-specific and non-specific brain organoids to opioids has contributed to our understanding of how POE can affect neuronal growth, survival, morphology, and function in the developing brain (Table 2). As is often wont to happen, however, these advancements have also brought to light caveats associated with the use of these cultures, gaps in knowledge, and areas for improvement.

One notable vacuum pertains to the absence of brain organoids or spheroids recapitulating the neurobiology of maternal OUD. To date, iPSCs and differentiated cultures have not been derived from pregnant women with dependent upon opioids or undergoing MAT for addiction. As mentioned in Section 2.2 above, the metabolism and pharmacokinetics of opioids are significantly altered in pregnant women, which manifests in the rapid clearance of these drugs and heightened dosages required to achieve the same effects as non-pregnant individuals (Farid et al., 2008; Costantine, 2014; Feghali et al., 2015). Therefore, an *in vitro* model that recapitulates these differences will be crucial to mechanistically understanding the pathobiology and progression of maternal OUD in the brain.

The accuracy and utility of organoids for studying maternal OUD (as well as POE) will be contingent upon the expansion of patient representation. Mirroring a historic problem in biological research, the comprehensive inclusion of female-derived iPSCs in studies of both OUD and POE has been sparse. Of the 11 articles using 3D models mentioned in this review, only five reported the application of female iPSC-derived cultures (Wu et al., 2020; Notaras et al., 2021; Ho et al., 2022; Nieto-Estévez et al., 2022; Strong et al., 2023) (Tables 1, 2). Even so, most of their major experiments were still conducted using male-derived iPSCs with limited numbers or utilization of female subjects. In one study, the cells were derived from a sole adolescent female (<18 years-of-age) (Strong et al., 2023). These omissions are noteworthy, since gender, age, and reproductive status have been shown to influence opioid pharmacodynamics and effects in the CNS (Zubieta et al., 1999, 2002; Lopes et al., 2021). Moreover, a large part of the increase in illicit opioid misuse over the past two decades has been in women of reproductive age (Ross et al., 2015). Considerations of putative differences in fetal brain development between females and males (De Lacoste et al., 1991; Studholme et al., 2020) are also essential for contextualizing the effects of POE. Moving forward, the implementation of female iPSC-derived 3D cultures will be of vital importance for dissecting this interplay of gender and opioid effects in studies of both OUD and POE. Leveraging clinically available somatic sources like blood plasma or urine (which also reflect opioid bioavailability) from pregnant women and neonates will further serve to supplement the feasibility, accuracy, and utility of these models.

Additional augmentation of these models may be achieved by the diversification of the (a) opioids and (b) brain-regions investigated. Regarding the former, few studies have expanded beyond assessing opioid-based pharmacotherapies for OUD (i.e., methadone and buprenorphine) or using opioid-receptor agonists with no clinical relevance (i.e., DAMGO, endomorphin) (Tables 1, 2). Given the likelihood of concomitant maternal and fetal exposure to other opioids inside and outside the clinic, it is crucial to widen the scope of future studies to probe the effects of non-MAT opioids with high misuse potential, such as oxycodone, fentanyl, and hydrocodone. Although such drugs have started to be included in studies of OUD and POE (Kim et al., 2021; Boutin et al., 2022; Ho et al., 2022), further work will be necessary to synchronously, asynchronously, or independently study their impact in the context of both conditions.

With respect to the latter issue, only three articles included in this review mention using 3D cultures to recapitulate brain regions outside of the forebrain (Kim et al., 2021; Fernandes et al., 2022; Strong et al., 2023) (Tables 1, 2). In future, the increased inclusion of midbrain, hindbrain, and brainstem organoid cultures may help provide greater insight into the mechanisms underlying OUD and POE (Tieng et al., 2014; Jo et al., 2016; Qian et al., 2016; Eura et al., 2020; Nickels et al., 2020; Valiulahi et al., 2021). The value of this approach is underscored not only by the involvement of these brain regions in the opioid addiction cycle, but also by prior efforts to use iPSC-derived midbrain dopaminergic or brainstem pre-Bötzinger Complex neurons to investigate the etiology or impact of OUD (Sheng et al., 2016b; Halikere et al., 2020; Guo et al., 2023).

In addition, further application of more complex organoid cultures (i.e., multi-region organoids, assembloids, vascularized organoids, and microglia-integrated organoids) may help dissect processes of neural patterning, neuronal migration, or neuroinflammation in the context of OUD or POE (Lancaster and Knoblich, 2014a; Abud et al., 2017; Bagley et al., 2017; Birey et al., 2017; Xiang et al., 2017, 2019; Cederquist et al., 2019; Ao et al., 2021; Hong et al., 2023; Zhang et al., 2023). Optimizing existing organoid protocols may also contribute to the body of knowledge surrounding opioid effects on glia. While brain organoids have regularly been reported to contain astrocytes and oligodendrocytes, cell-type proportions have varied. Generating region-specific cultures with consistent ratios of such cell types along biologically relevant timelines, as done by Strong et al. (2023), will be crucial to further anatomizing how opioids affect gliogenesis, astrogliosis, and myelination. Furthermore, studies of opioid activity and dynamics may also be expanded to include CNS components outside of the brain. This may be done through bioengineered platforms for organoid generation, such as those recapitulating the spinal cord and blood–brain barrier (Brown et al., 2020; Cai et al., 2023).

Finally, as models of maternal OUD and POE advance, it will be important to address challenges associated with the biology of organoid technology itself. A persistent complication is the immaturity of iPSC-derived differentiated tissues, which muddles the interpretation of adult disease neuropathology. Notably, human iPSC-derived neurons and brain organoids have been observed to developmentally correspond to embryonic or fetal maturation, having undergone a process of transcriptional and epigenetic “rejuvenation” or “erasure” upon cellular reprogramming. Although restricted by the absence of biological systems like the blood brain barrier or placenta, both of which play an important role in opioid dynamics, the immaturity of these culture systems proves advantageous for antenatal studies of perturbagens like opioids. Insights from such *in vitro* investigations serve to compliment *in vivo* studies of POE. However, this same characteristic makes the *in vitro* recapitulation of adult OUD and the extrapolation of advanced neurological consequences difficult. Nevertheless, recent evidence that CpG sites contributing to age-related morbidity and mortality are maintained following stem cell induction has opened an avenue in service of this aim (Mendez et al., 2023). Future studies may leverage this knowledge to propel brain organoid maturation and improve the technology’s application for the analysis of maternal OUD *in vitro*. As it stands, organoid technology remains a constructive tool to study the neurodevelopmental pathogenesis and progression of this disorder.

Considering these outstanding challenges, the usage of iPSC-derived brain organoids and spheroids to model OUD and POE seems to be in its proverbial infancy (McNeill et al., 2020; Niemis et al., 2023). Moving forward, it will be necessary to expand research beyond just using 3D cultures as platforms for opioid screening and testing, as has been the status quo. This approach has meant that any insight into the neuropathological underpinnings or consequences of OUD and POE has often been an incidental byproduct. As many of the articles summarized in this review demonstrate, however, directly using organoids and spheroids to model OUD and POE is an indispensable technique. This focus has already helped make meaningful headway in understanding the neurobiology of these conditions and has established a solid bedrock upon which future studies may be built. As it stands, this progress is exceedingly necessary, given the severe, long-lasting impacts of maternal OUD and fetal POE at the individual and societal level. iPSC-derived organoid technology provides a unique opportunity for rapid, targeted innovation in this field, not only to understand the causes and consequences of OUD and POE, but also to explore crucial avenues for their remedy.

## Author contributions

ID: Conceptualization, Visualization, Writing – original draft, Writing – review & editing. GGH: Conceptualization, Funding acquisition, Supervision, Writing – review & editing.
